# Our Treatment Strategy for Critical Limb Ischemia

**DOI:** 10.1155/2013/437471

**Published:** 2013-12-10

**Authors:** Tetsuo Yamada, Kiyoshi Onishi, Makoto Utsunomiya, Masato Nakamura

**Affiliations:** ^1^Department of Plastic and Reconstructive Surgery, Toho University Ohashi Medical Center, 2-17-6 Ohashi Meguro-ku, Tokyo 153-8515, Japan; ^2^Department of Cardiovascular Internal Medicine, Toho University Ohashi Medical Center, 2-17-6 Ohashi Meguro-ku, Tokyo 153-8515, Japan

## Abstract

For the treatment of critical limb ischemia, collaboration with wound specialists and cardiologists performing revascularization is important. The foot care unit affiliated with related departments opened at our hospital in July 2010 for limb salvage, mainly under the leadership of the departments of cardiovascular internal medicineand plastic surgery. We have treated 194 patients up until October 2012. The primary diseases included 81 cases (87 limbs) of foot ulcer and gangrene, with complications of peripheral arterial diseases (PADs) in all cases. Intravascular treatment was conducted for 69 limbs with PAD complications, and the initial success rate was 85.5%, of which *surgical* debridement or minor amputation was performed on 32 limbs. Regarding open wounds following operation and chronic ulcer, platelet-rich plasma therapy was conducted in 29 limbs and negative pressure wound therapy in 15 limbs. Among all of the patients treated, 58 limbs healed, 10 cases died, and the others are currently receiving ongoing treatment. Cardiovascular internal medicine specialists and plastic surgeons examine patients together at the outpatient clinic and prepare and implement a multidisciplinary treatment plan including vascular reconstructions and operation. We cooperate with physicians in each related department and efforts in team medicine have been made for the purpose of limb salvage.

## 1. Introduction

In Japan, as the number of patients with diabetes mellitus and patients undergoing dialysis increases, the number of patients with peripheral arterial disease (PAD) or critical limb ischemia (CLI) is also increasing. In the treatment of CLI accompanied by ulcer or necrosis, treatment of peripheral arteries as well as treatment of ulcers and necrotic tissues is required [1]. Therefore, cooperation of cardiologists performing revascularization and wound reconstructive surgeons is essential. The foot care unit affiliated with related departments opened at our hospital in July 2010 for limb salvage, mainly under the leadership of the departments of cardiovascular internal medicine and plastic surgery. Treatment with minimal invasion is desirable for patients with CLI; in a large proportion of patients with CLI, it is difficult to perform surgery because of complications; there are an insufficient number of vascular surgeons who perform peripheral bypass surgery; and because of these reasons, our hospital has performed endovascular treatment (EVT) as the first option. In recent years, EVT has made remarkable progress, and it can be performed repeatedly with minimal invasion in patients in whom bypass surgery is not indicated because of reasons such as poor systemic condition; therefore, endovascular treatment has been increasingly performed in many patients. In the United States of America, bypass surgery has been actively performed for a long time; however, it has been reported that the proportion of EVT has increased gradually and that the proportion of EVT exceeded that of bypass surgery in 2005 [2]. For treatment results as well, the BASIL trial [3] which compared EVT and bypass surgery showed that there was no significant difference in salvage rate of lower limbs or mortality rate within 3 years. Especially, the region of the lower leg arteries is mainly positioned as the region for prevention of amputation and reduction of amputation in CLI, and revascularization of lower leg arteries is essential for salvaging lower limbs. It has been reported that there is no difference in limb salvage rate between EVT and bypass surgery, but that a long-term patency cannot be expected by EVT, and that a patency of only 1 of 3 branches of the lower leg will achieve only limited blood flow [4]. Therefore, in wound management after EVT, evaluation of blood flow by wound reconstructive surgeons is important. In addition, we actively select negative pressure wound therapy (NPWT) and platelet-rich plasma (PRP) therapy for postoperative open wounds and ulcers with delayed wound healing with an understanding that it is important to promote wound healing and shorten the duration of treatment by various adjuvant therapies. In this report, we describe our treatment policy for patients with CLI.

## 2. Materials and Methods

**Figure 1:**
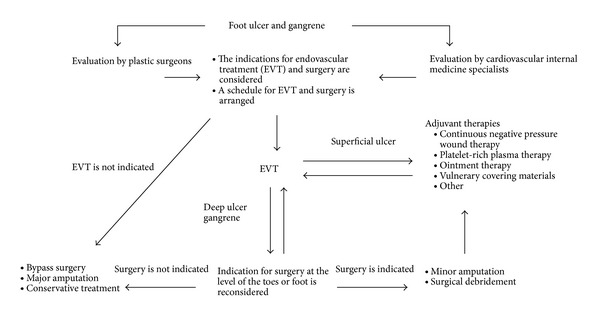
Treatment algorithm for critical limb ischemia.

From July 2010 to October 2012, a total of 194 patients (120 men and 74 women) visited the foot care outpatient clinic of our hospital; the age of the patients ranged from 31 to 99 years (mean: 72.1 years). At first visiting our hospital, a cardiovascular internal medicine specialist and a plastic surgeon examined the patient together and confirmed the presence or absence of ischemic clinical findings such as cold feeling, numbness, intermittent claudication, and pain at rest with pulsation of the dorsalis pedis artery and posterior tibial artery. In the examination of the local wound site, attention was paid to the presence or absence of wound infection; if osteomyelitis or necrosis of the subcutaneous tissue is suspected, objective evaluation was performed by CT, MRI, and so forth. Causes of ulceration of lower limbs include venous ulcer, neurogenic ulcer, ulcer due to collagen disease, traumatic ulcer, and infectious ulcer; it was determined whether ulceration of lower limbs was due to ischemia rather than these diseases. Especially, for patients with diabetes mellitus and dialysis patients, the wound was often due to complex pathological conditions; therefore, evaluation of the underlying disease and thorough evaluation of the systemic condition were performed at the same time. If complication by PAD is suspected, evaluation of skin perfusion pressure (SPP), ankle brachial pressure index (ABI), and ultrasonography of lower limb arteries was performed for the evaluation of ischemia. For patients who received a diagnosis of CLI, revascularization was performed with EVT by the department of cardiovascular internal medicine as the first choice, and a treatment plan was established and implemented in accordance with our treatment algorithm (Figure 1).

## 3. Results

**Figure 2:**
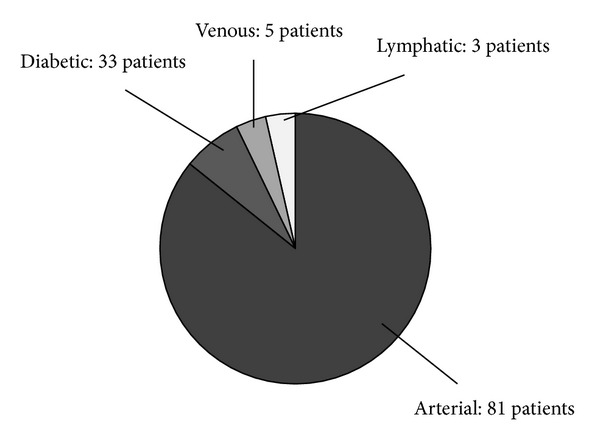
Cause of ulcer of lower limbs in 122 patients who visited the foot care outpatient clinic.

By October 2012, 122 patients with ulceration of lower limbs visited the foot care outpatient clinic; of these, there were 81 patients with CLI accompanied by skin ulcer or gangrene of the foot (Figure 2). The rate of complication by diabetes mellitus in patients with CLI was 45.1%; there were a total of 35 dialysis patients (24 patients complicated by diabetes mellitus and 11 patients not complicated by diabetes mellitus). EVT was performed in 69 of 87 limbs in 81 patients with CLI; the initial success rate was 85.5% (59 of 69 limbs). In all of the 10 limbs in which restart of blood flow was not achieved, the site on which EVT was performed was the lower leg region; of those, 4 patients (4 limbs) were referred to another hospital for surgical bypass. Minor amputation or debridement was performed on 32 limbs after EVT, and major amputation was performed on 7 limbs of all patients with CLI. For open wounds after surgical procedure following EVT and ulcers with delayed wound healing, in addition to regular ointment treatment (sucrose, tretinoin tocoferil, sulfadiazine silver, alprostadil alfadex, etc.), PRP therapy was performed on 29 limbs, and NPWT was performed on 15 limbs. The treatment course of all these patients with CLI was as follows: cured: 58 limbs; under treatment: 11 limbs; death: 10 patients (18 limbs).

### 3.1. Case Study

**Figure 3:**
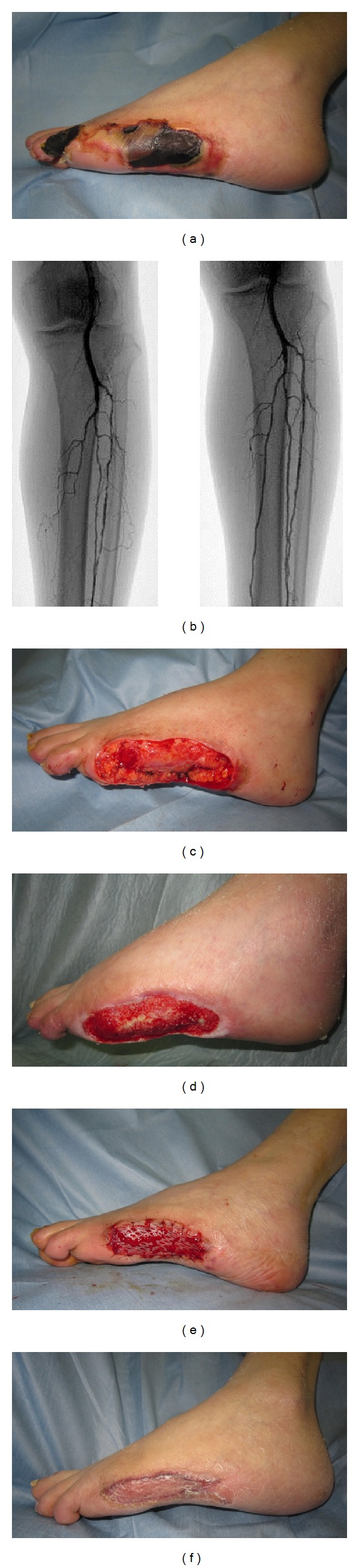
(a) Findings at first visiting our hospital: gangrene of the left 4th toe and skin necrosis of the lateral foot were observed. (b) Angiographic findings of the leg (left: before EVT; right: after EVT): occlusion of the left posterior tibial artery and stenosis of the anterior tibial artery and peroneal artery were observed; therefore, the 3 branches were dilated with a balloon. (c) Findings immediately after amputation of the left 4th toe and debridement: the left 4th toe was amputated at the metatarsal shaft, and skin necrosis of the lateral foot was removed. (d) Finding at the end of NPWT treatment using the V.A.C. ATS therapy system was performed for 3 weeks, and granulation was observed. (e) Finding immediately after split-thickness skin grafting: a skin graft with PRP (platelet-concentration ratio: 5.1x) was performed. (f) Finding at 2.5 months after the treatment.

An 84-year-old man visited our hospital with gangrene of the left 4th toe and skin necrosis of the left lateral foot. He was receiving insulin therapy for diabetes mellitus and had a history of amputation of the left 5th toe. Four weeks ago, an ulcer of the left 4th toe was formed by a blister. He visited a hospital and received conservative treatment; however, necrosis enlarged; therefore, he was referred to the foot care outpatient clinic (Figure 3(a)). The results of blood chemistry were as follows: CRP: 0.38 mg/dL; WBC: 9700/*μ*L; HbA1c (JDS): 7.4%; wound culture test was negative. It was impossible to measure SPP because of pain. ABI was 0.88; ultrasonography of lower limb arteries showed stenotic lesions scattered in the anterior tibial artery and peroneal artery, and there were occlusions of the posterior tibial artery. Plain CT scan of the left foot showed no obvious findings suggestive of osteomyelitis. Local irrigation and treatment with ointment were performed; on day 7 after presentation, EVT was performed. In EVT, 3 branches were dilated with a balloon (Figure 3(b)). After EVT, ABI was 0.99 and SPP around the wound was 28 and 36 mmHg. On day 8 after EVT, amputation of the left 4th toe and debridement were performed under general anesthesia (Figure 3(c)). After surgery, obvious extension of necrosis was not observed; on day 5 after surgery, NPWT was performed continuously for 3 weeks with a pressure of 125 mmHg (Figure 3(d)). Satisfactory formation of granulation tissue was observed; therefore, split-thickness skin grafting added PRP was performed on day 32 after surgery (Figure 3(e)). The skin graft showed complete survival, and a cure was observed at 2.5 months after the treatment (Figure 3(f)).

## 4. Discussion

### 4.1. Treatment Algorithm for CLI

After evaluation of the blood flow in lower limbs and the wound and thorough evaluation of the systemic condition, indications for EVT and surgery are considered. In patients with CLI accompanied by a wound, basically, priority is given to revascularization, and wound management is performed after confirming that sufficient blood flow to the wound is obtained. In patients who require surgery, it is important to give priority and schedule EVT and surgery. Indication for surgery and surgical methods are considered based on the effect to improve SPP and angiographic findings and the angiosome after EVT; therefore, priority is given to EVT as much as possible, and surgery is planned within 2 weeks after EVT. However, in patients with severe infection who undergo drainage or have cellulitis and show high levels of CPR and WBC in blood chemistry, there is a risk that infection may spread because of improvement of blood flow [5]. For patients in whom infection subsided by conservative treatment such as local irrigation and drip infusion of antibiotics, surgery is planned on the following day after EVT. For patients in whom infection does not subside by such treatment, priority is given to surgery, and the schedule is arranged so that EVT will be performed within a few days after surgery. If surgery is performed on the following day after EVT, SPP may not be increased immediately after EVT; therefore, indication for surgery and surgical methods is determined based on angiographic findings of the foot after EVT. For patients in whom it was considered possible to perform surgery, debridement or minor amputation is performed; for open wounds after surgery, adjuvant therapies such as regular ointment therapy, use of vulnerary covering materials, NPWT, and PRP therapy are selected. If it is considered that there is insufficient blood flow to the wound after EVT, a repeat EVT is considered. Patients in whom it is considered impossible to perform EVT or surgery after EVT are referred to appropriate departments, where indication for bypass surgery or major amputation is considered; for patients in whom surgery is not indicated, conservative treatment is selected (Figure 1).

### 4.2. Wound Management after EVT

In CLI with a wound in the foot, blood flow below the ankle region plays an important role in wound healing; it is difficult to prevent amputation by treatment of the proximal side alone, and catheterization of the foot is required to prevent amputation [6]. Revascularization considering the angiosome is important [7]; therefore, in our hospital the plastic surgeon who performs wound management participates in EVT as much as possible and preferentially identifies blood vessels to be dilated. It has been reported that there is no difference in limb salvage rate between EVT and bypass surgery, but that a long-term patency cannot be expected by EVT, and that a patency of only 1 of 3 branches of the lower leg will achieve only limited blood flow [4]. Therefore, in wound management after EVT, evaluation of blood flow is important. If delay in wound healing or a decrease in local peripheral circulation detected by SPP is observed, restenosis is suspected, and evaluation of blood flow and repeat EVT are considered. SPP monitors blood flow at a depth of about 1.5 mm from the skin surface, and the efficacy of SPP for evaluation of ischemia has been reported [8]. On the other hand, Komoda maintains that it is important to select a treatment method without placing too much importance on apparent SPP values, after considering the change in blood circulation due to collateral vessels, AV shunt due to peripheral nerve disorder, venous stasis, surgical procedures, and so forth, and after evaluating the pathological conditions of the lower limb with various changes [9]. Our hospital also considers SPP as an indicator for selecting a treatment policy; however, we consider that clinical symptoms are the most reliable indicators, and we try to monitor the wound as much as possible. In addition, we actively select NPWT and PRP therapy with an understanding that it is important to promote wound healing and shorten the duration of treatment by various adjuvant therapies. NPWT is a wound care system initiative reported by Argenta and Morykwas [10] in 1997; it promotes a cure by locally applying negative pressure to the wound. Recently, the efficacy of NPWT has been widely recognized, and NPWT has been applied to the treatment of open wounds and intractable ulcer including pressure sore; in 2012, Kasai et al. reported that NPWT was also effective for patients with CLI whose SPP was not more than 30 mmHg [11]. PRP has attracted attention since 1998 when Marx et al. [12] reported that they used PRP for bone grafting in the field of dentistry; currently, the efficacy of PRP for the treatment of various pathological conditions is being recognized. PRP is plasma which has the expected effect of promoting wound healing with growth factors (located in the *α*-granules of platelets and effective for wound healing and tissue regeneration), which are highly concentrated and degranulated. Recently, there have been some clinical reports in the field of plastic surgery as well, and the efficacy of PRP in the treatment of chronic ulcer has been widely known [13]. In 2011, Bir et al. infused PRP in mice with diabetic ischemic limbs, histologically investigated the vascular regeneration effect of PRP, and reported that a vascular regeneration effect of PRP, which was superior to that in the control group, was observed [14]. In our experience so far, NPWT is indicated for postoperative open wounds, and PRP is used for small postoperative open wounds and ulcers with delayed wound healing; we have achieved favorable treatment results.

### 4.3. Future Challenge

The TASC classification [1] shows guidelines for which EVT or surgical bypass should be the first option for revascularization, based on the lesion classification of arteriosclerosis obliterans. Treatment with minimal invasion is desirable for patients with CLI; in a large proportion of patients with CLI, it is difficult to perform surgery because of complications; there are an insufficient number of vascular surgeons who perform peripheral bypass surgery; and because of these reasons, our hospital has performed EVT as the first option. However, it is difficult to dilate highly calcified lesions and long occlusive lesions by EVT, and EVT is not indicated for patients with decreased renal function or allergic contrast agent. Patients with a large tissue defect require a large amount of blood flow; in such patients, surgical bypass is more effective [4]. Therefore, there is a limitation on limb salvage with EVT alone, and collaboration with vascular surgeons is essential. We consider this issue as a future challenge. However, it is difficult for specialists in different fields required for treatment to be in a facility at the same time; in the future, we hope to establish a medical practice model based on a collaborative relationship in the community and between facilities.

## 5. Conclusion

We reported on the foot care medical system of our hospital, which consists mainly of cardiovascular internal medicine and plastic surgery, and our treatment policy for patients with CLI. Main features of the team approach of our hospital are that the plastic surgeon participates in EVT as much as possible and identifies blood vessels to be dilated and aggressively performs NPWT and PRP therapy for postoperative open wounds and ulcers with delayed wound healing to promote wound healing and shorten the duration of treatment. We aim to provide team medical care for less invasive and safer treatment with a shorter duration by timely collaboration with related departments. In addition, we would like to establish a medical practice model based on a collaborative relationship in the community and between facilities, which is a future challenge for us.
